# The sustainability of an Australian initiative designed to improve interdisciplinary collaboration in mental health care

**DOI:** 10.1186/1752-4458-7-10

**Published:** 2013-03-19

**Authors:** Kylie King, Jo Christo, Justine Fletcher, Anna Machlin, Angela Nicholas, Jane Pirkis

**Affiliations:** 1 Centre for Health Policy, Programs and Economics, Melbourne School of Population and Global Health, University of Melbourne, Victoria 3010, Australia

**Keywords:** Mental health, Interdisciplinary, Sustainability, Networks

## Abstract

**Background:**

The Australian Mental Health Professionals Network (MHPN) is fostering a collaborative, interdisciplinary approach to mental health care through the establishment of local interdisciplinary networks of mental health professionals. This paper reports on those factors seen by MHPN participants and staff as having affected the formation and continuation of interdisciplinary networks, and therefore the likely sustainability of these groups.

**Method:**

The paper draws on qualitative data from focus groups with mental health professionals participating in MHPN activities and MHPN staff.

**Results:**

The findings suggest that MHPN’s approach to establishing sustainable interdisciplinary networks has been influenced by a number of factors at the micro-, meso-and macro levels. At the micro-level, factors such as clarity and structure of ongoing meetings, individual dynamics and the role of ‘champions’ can promote or constrain sustainability of ongoing networks. Those networks that had established following an initial workshop and had continued to meet as an interdisciplinary network tended to be led by well-respected co-ordinators, involve members who are enthusiastic and keen to learn from each other, have a flexible structure and meet regularly for a well-defined purpose. These features are underpinned by good communication between network members and with MHPN administration. At the meso- and macro-levels, the key issue relates to resourcing, as well as the wider policy context.

**Conclusions:**

The support and practical resources provided by MHPN have been crucial in guiding successful networks as they form and continue to meet on a regular basis. The networks have also required internal leadership and support, and a clear purpose in order to form and to continue their activities. These findings are consistent with the literature, which states that sustainability of programs is reliant on factors at the project design and implementation level, as well as on factors inherent within the host organization and at the wider community level.

## Background

There is increasing recognition that interdisciplinary mental health care can often lead to better outcomes for consumers than care which is provided by individual professionals working in relative isolation in private rooms [1,2]. The two underlying elements of collaborative care are the construction of collective action as a method of meeting complex client needs and the integration of different professional perspectives that promote relationships which are respectful and trusting [3]. However, collaborative interdisciplinary teams are not common in Australian primary health care, and collaborative care is made difficult by boundaries between professionals and within health services [4]. Understanding of the complexity of relationships between health professionals is limited [3].

Establishing the kind of relationships necessary to underpin such collaborative care can be difficult, and sustaining them can be even harder [1]. Maintenance of these relationships can be promoted at the individual (e.g., education to promote individual change in behaviour), organizational (e.g., leadership, program champions), community action (which creates new partnerships) and system levels [5]. Often, co-ordinated leadership and external support are needed [6]. The current paper describes an Australian initiative known as the Mental Health Professionals Network (MHPN) that is fostering a collaborative clinical approach to the provision of mental health care by promoting interdisciplinary communication and networking between psychiatrists, general practitioners (GPs), psychologists, mental health nurses, social workers, paediatricians and occupational therapists, with the ultimate aim of improving consumer outcomes.

MHPN is funded by the Australian Government’s Department of Health and Ageing and began operating in August 2008. MHPN rolled out a series of workshops across Australia that were facilitated by one local mental health professional (most commonly a psychologist) and attended by a mixed group of other mental health professionals from the same geographical area. These workshops were supported by a range of educational materials, a website and web portal (MHPN Online) and a toll-free telephone information line. Through informal networking and the presentation of case studies, the workshops aimed to enhance understanding of how and what each profession contributes to the care of the consumer and to improve mental health professionals’ skills in supporting people with mental illness [7]. By July 2010, MHPN had successfully run 1,156 workshops, which were attended by 11,930 mental health professionals [8,9].

The workshops also aimed to generate interdisciplinary networks, defined as “groups of local mental health professionals around the country who meet regularly to network and discuss mental health issues and thus facilitate improvements in the collaborative delivery of mental health care” [7]. Unpaid volunteer network co-ordinators guide the directions of the network and attend to logistical tasks associated with organising meetings. MHPN staff provide administrative support and guidance to the network co-ordinators. MHPN also makes $500 available per network per annum to assist with network maintenance. MHPN Online supports ongoing networking and interdisciplinary collaboration through a members search function, a networks search function, clinical and general discussion forums, a mailbox that allows MHPN Online members to communicate through personalised emails, event organisation tools, and help pages.

By July 2010, 938 of the 1,156 workshops (81%) had individually or jointly formed a total of 705 ongoing networks [8-10]. The sustainability of these interdisciplinary networks increasingly became a focus of MHPN activities throughout 2009 and 2010. It became apparent that more efforts and resources needed to be invested to ensure the continuity of these networks.

Looking at the sustainability of any program is important [11-15]. Although new programs are often based on the assumption that if they meet an identified need and prove to be effective they will continue to exist, this is not always the case. Many new programs are not sustained beyond the first few years after termination of initial funding [13], and this can diminish community support for new programs following past patterns of program funding withdrawal [14]. Within this evaluation of MHPN network sustainability, ‘sustainability’ was defined as a multidimensional concept of program continuation which can be considered in relation to components such as project design and implementation, the host organization and the wider community context, as outlined in the literature [13,14]. Project design and implementation factors include program financing and resourcing, as well as program flexibility, communication of successes and evaluation [15]. Organizational factors which influence sustainability include structure, program champions and integration with existing programs, and wider community factors influencing sustainability such as community support [14]. The process of sustainability includes the concepts of time and permanence but does not limit these concepts. That is, sustainability does not impose timeframes on stages or processes. Our definition of sustainability also does not infer a static program; rather, it suggests an adaptable and fluid program where successful elements are developed and less successful elements reworked [11]. This adaptability is particularly important within the large scale nature of the MHPN initiative, which was evolving over time.

We were commissioned to conduct an evaluation of MHPN that explicitly considered network sustainability. The evaluation is reported in full elsewhere [8,9]. The MHPN evaluation timeframe required that the evaluation of network sustainability occur at a time-point two years after the beginning of MHPN’s activities. While a later evaluation would be needed to establish whether the interdisciplinary networks formed at the time of the evaluation were sustainable in the longer-term (for example, several years beyond their inception), our evaluation focused on those networks that had already formed and who had continued to meet for the purpose of interdisciplinary collaboration beyond an initial meeting. This paper focuses on issues deemed by MHPN staff and participants as important in affecting the formation and continuation of networks that had continued to meet on a regular basis for the purpose of interdisciplinary collaboration.

## Method

Eleven focus groups were undertaken: ten with mental health professionals who had attended MHPN events (December 2009 and April 2010) and one with MHPN staff (May 2010). Focus group participants were asked to consider barriers to, and enablers of, ongoing interdisciplinary networks. By including a focus group component in the broader evaluation, we hoped, as argued by Willis, Green, Daly, Williamson and Bandyopadhyay (2009), that a range of insights might be provided that might not be gleaned from the other evaluation methods used, and that we would also gain an understanding of how and why perspectives might differ between individual professionals, professions and geographic locations. Specifically, opinions were sought on the challenges within and between professional groups in undertaking interdisciplinary networking.

Ethics approval was obtained from the University of Melbourne’s Human Research Ethics Committee.

### Mental health professionals focus groups

#### Focus group recruitment strategy

MHPN provided researchers with a de-identified list of workshop attendees for each of the proposed ten focus group locations: urban New South Wales; urban and rural Victoria; urban and rural Western Australia, urban and rural Queensland, urban South Australia, one in the Northern Territory and one in Tasmania (rural). Locations of the focus groups were chosen in conjunction with MHPN and in relation to where workshops had already been conducted. To achieve the recruitment goal of 10 to 12 participants per focus group, with a mix of professions in each group, a stratified random sampling technique was used. Namely, a random sample of seven individuals was chosen from each core profession in each location to be invited to participate. This figure was chosen based on an anticipated 30% response rate. This recruitment strategy was adopted to try to ensure that all of the professions were represented in each focus group rather than basing it on the relative proportions of each of the professions who had attended workshops. This required over sampling of some of the professions under-represented at workshops (i.e. psychiatrists, GPs, social workers, OTs) and under- sampling of psychologists, who are heavily represented at workshops. Where seven or less individuals from a profession had attended a workshop in the location, all were invited to participate in that focus group.

The researchers then provided the MHPN project officer with the ID numbers of each of those attendees randomly selected to be invited. The MHPN sent these individuals an email containing the evaluation plain language statement and consent form and a request to fax the consent form to the researchers if they wished to take part in the focus group.

An email was sent by the researchers on receipt of participants’ consent forms asking participants to confirm their attendance. Ten days later, additional attendees were invited to each focus group for those professions that had not yet been adequately represented or in locations where few people had consented overall using the random sampling technique described above. This was to maximise the chance of getting adequate representation of each professional group. A second round of invitations was sent out a week after the reminder email. Additional attendees were invited to each focus group for those professions that had not yet been adequately represented or in locations where few people had consented overall.

#### Focus group participants

In total, 481 MHPN workshop attendees were invited to take part in a focus group, 89 of whom then participated in focus groups in December 2009 and April 2010, representing an 18% response rate. Focus groups lasted for 60 to 90 minutes and were held on a weeknight, out of office hours. Table 1 presents the demographic profile of focus group participants. Of those invited, psychologists (29%) and social workers (24%) were most likely to participate and psychiatrists were the least likely to participate (7%). Focus group participants had attended between one and six initial workshops, and one-third were involved in an ongoing network. Eighteen percent of participants were not yet involved in an ongoing network.

**Table 1:** Demographic profile of MHPN focus groups by location

	**Location**										
	**Vic. Urban**	**Vic. Rural**	**WA Urban**	**WA Rural**	**NSW Urban**	**Tas. Rural**	**NT Rural**	**SA Urban**	**Qld Urban**	**Qld Rural**	**Overall**
Profession											
General practitioner	1	0	1	1	0	1	1	2	0	3	10
Psychologist	1	4	1	0	2	4	2	2	0	1	17
Psychiatrist	1	0	2	1	0	0	1	1	1	0	7
Social worker	1	0	1	0	2	2	0	0	1	2	9
Mental health nurse	1	4	3	5	3	1	2	1	3	3	26
Occupational therapist	6	1	3	2	4	1	0	1	1	1	20
Gender											
Male	2	2	3	2	6	3	1	3	0	3	25
Female	9	7	8	7	5	6	5	4	6	7	64
Number of MHPN initial workshops attended											
Mean	2.09	2.11	1.45	1.44	1.45	3.56	1.83	1.57	1.67	2.1	1.927
Standard deviation	1.3	0.6	0.93	0.73	0.69	1.67	0.75	0.98	1.21	0.99	0.985
Range	1-5	1-3	1-4	1-3	1-3	1-6	1-3	1-3	1-4	1-4	1-6
Involvement in ongoing networks											
Yes	3	7	4	1	2	1	3	1	3	5	30
No	3	2	6	3	7	8	3	6	4	1	43
Not yet	5	0	1	5	2	0	0	0	3	0	16

### MHPN staff sustainability focus group

The focus group with MHPN staff involved Network Sustainability Project Officers and Senior Project Officers. The views of these individuals were considered important because their provision of administrative support and guidance to the networks was instrumental in developing and maintaining the networks. All nine staff were emailed an invitation, and eight participated.

Further details about the focus groups are shown in Table 2. The focus groups were structured, but they were also fluid, with little probing from the evaluators. Interactions between participants were encouraged with participants’ non verbal cues observed by facilitators to help guide discussion. The focus groups were each attended by more than one evaluator. Some evaluators acted as scribes, focusing on and making written notes about the dynamics and process of the group, while the other evaluators acted as facilitators, focusing on the content of the group.

**Table 2:** Focus group questions

	**Mental health professionals**	**MHPN staff**
Duration	60-90 minutes	90 minutes
Questions	What is your profession?	Are mental health professionals seeking ongoing networks?
	How many MHPN workshops and networks have you attended?	How are ongoing networks being coordinated and who is involved?
	What has your experience been of interacting with other mental health professionals?	What are the barriers to ongoing networks?
	How well have MHPN workshops facilitated interdisciplinary collaboration?	What are the enablers of ongoing networks?
	How have MHPN workshops provided opportunities to build interdisciplinary networks?	Describe the best example of an ongoing network.
	Has attending a MHPN workshop had an impact on your practice and client outcomes?	
	How could the MHPN better engage health professionals who are less likely be involved in networking activities?	
	How do the MHPN aims for interdisciplinary collaboration and networking match with your needs?	

We undertook directed content analysis [16] of the verbatim focus group transcripts . This involved two evaluators identifying a set of key themes and producing a template to organize these themes into a coded hierarchy. Higher order themes were developed by clustering lower order codes. This approach allowed the flexibility of some themes being developed a priori and others being developed during the analysis process. Initial themes were based on the focus group questions. Themes identified during the analysis process were then based on the content of the focus groups and the interactions between group members. The final thematic focus group schedules are shown in Table 3.

**Table 3:** Thematic focus group template used to guide analysis

**Mental health professionals**	**MHPN staff**
Previous interaction with other mental health professionals:	Are mental health professionals seeking ongoing networks?
i. Collaboration	i. What evidence substantiates this response
Intra-disciplinary	
Inter-disciplinary	
ii. Systemic issues	
Supportive structures	
Unsupportive structures	
How well have MHPN workshops facilitated collaboration?	How are ongoing networks being coordinated and who is involved?
i. Individual motivators	i. Role of the co-ordinator
Referrals	ii. Role of MHPN staff
Networking	
Collaborative learning	
Knowledge of other professionals’ roles	
ii. Workshop style/format	
iii. Facilitator factors	
iv. Disciplinary mix	
v. MHPN resources	
How well have interdisciplinary networks been built?	What are the barriers to ongoing networks?
i. Ongoing resource issues	i. MHPN resources
Role of co-ordinator	ii. Role of the co-ordinator
Funding	iii. Role of MHPN staff/input of MHPN
Workshop structure	iv. Purpose of network
Clarity of purpose	v. Clarity of what works
ii. Individual factors	vi. Content of network meetings
iii. Other suggestions	vii. Local issues
Need for a Supporting structure (i.e. MHPN)	viii. MHPN online
Need for local model	ix. Professional mix
Topic/interest based vs. location based	
Impact on practice and client outcomes:	What are the enablers of ongoing networks?
i. No impact	i. MHPN resources
No change in practice	ii. Role of the co-ordinator
Too soon	iii. Role of MHPN staff/input of MHPN
Difficult to measure	iv. Purpose of network
ii. Impact	v. Clarity of what works
Awareness within/between disciplines to refer to/consult	vi. Content of network meetings
	vii. Local issues
Awareness of services in public/private sector	viii. MHPN online
Understanding of professional roles/skills	ix. Professional mix
Understanding of system/processes	
Isolation/communication/collaboration with other professionals	
Communication to other professionals (incl. reporting)	
Skills	
Client outcomes	
How could the MHPN better engage health professionals who are less likely to be involved in networking activities?	Describe the best example of an ongoing network
i. Incentives	i. Processes
Food	ii. Strategies
Financial reimbursement	iii. Systems
Professional development points/training	iv. Resources
ii. Marketing	
By whom/to whom?	
Methods?	
Local understanding of services	
iii. Workshop structure	
Mediums	
Format	
Purpose	
Timing	
How do the MHPN aims for interdisciplinary collaboration and networking match with your needs?	
i. Ongoing resource issues	
Government policy	
MHPN funding/support	
ii. Individual factors	
Motivations	
Older vs. newer practitioners	
Public vs. private sector		

## Results

The findings from the focus groups revealed factors at the micro level (within network), meso level (interactions between networks and MHPN), and the macro level (broader mental health care context). This is consistent with results from other studies which have explored inter-professional approaches to collaboration [17,18].

### Micro-level factors

#### Network co-ordination

Most MHPN staff and mental health professionals commented on the need for ongoing co-ordination of the networks. Staff commented on the requirement of a “*champion co-ordinator”*: an individual who was a *“good leader”,* who was *“respected”* and had *“skill, competency, time, [and was prepared to put in] effort”* in order to foster network sustainability.

However, both MHPN staff and most mental health professionals also expressed some concerns about the sustainability of such a model of co-ordination. Staff feared that networks could fail when that individual gets *“overwhelmed”* and *“burnt out”,* and that a model of shared co-ordination by a number of individuals was likely to be more sustainable, but that this also presented challenges in terms of individuals working effectively together.

Both MHPN staff and mental health professionals also spoke about the difficulties in finding appropriate individuals to be involved in network coordination. The majority of mental health professionals suggested that participants were not always prepared to take on responsibility for co-ordinating a network without external funding or support:

“*You’re looking around everybody, do you want to do this again and everybody is nodding enthusiastically and you think, yes but, like I don’t want to organize, I don’t want to organize something else.”*

Some staff noted that a lack of *“skills and competencies”* in a co-ordinator can be a barrier to creating successful ongoing networks. In most mental health professional focus groups, participants also commented that having someone designated to fulfill the role who was “*funded, educated and supported”* was likely to be more viable.

#### Balancing the need for clarity of purpose and structure against flexibility

Mental health professionals reported some tension between a desire for greater clarity about the purpose of the networks and a desire for flexibility which enables each network to structure itself in the manner that best suits its particular needs. For many, the corollary of this was that potential network members struggled with how to move forward and organize and maintain the ongoing network. The following comment exemplifies this:

“There didn’t seem to be a clear defined plan … and while it was talked about being sustainable there was no sense of how it was going to be sustainable.... It was sort of left open for what to happen next.”

Some of the mental health professional focus group discussions also suggested that having a clear purpose could motivate people to attend and ensure that what they were doing was meaningful. This is illustrated in the following comment:

“I don’t see the point in having thousands of people meeting about thousands of things … I think we need goals, I think we need methodologies for achieving those goals and I think we need to set curricula before we actually sit down and start doing whatever we are doing.”

MHPN staff agreed that lack of purpose could be a barrier to success. They talked of their frustration at not being able to give networks clear guidance and instruction:

“We are not really clear about what makes a successful group and what they should be focusing on, and we are still working on that and clarifying that for ourselves.”

#### Format of meetings

MHPN staff and mental health professionals considered the format of the ongoing network meetings. Clarity of purpose, regularity, consistency and advanced organization of meeting dates and topics were viewed positively. These features were seen as important for enabling mental health professionals to plan ahead.

With respect to content, giving people opportunities to learn something new was seen as an attraction, and having an expert speaker was seen by some mental health professionals as a good way to achieve this.

Having meetings based around specific topics was also seen as a drawcard. Many suggested that it would be more relevant for them for networks to be grouped according to clinical specialties or particular topics rather than location.

#### Network structure: Open versus closed networks

Mental health professionals discussed whether networks should continue with the same practitioners that attended initial meetings or whether they should be open to anybody. In general, participants indicated that they would prefer groups to be open to allow people to move from group to group and to enable them to meet new people to expand their networking possibilities. This sentiment is exemplified by the following quotation:

“I’ve been actively avoiding becoming part of a closed group simply because one of the things that has worked really well for me in attending a large number of these groups is getting to meet a larger number of the mental health professionals within the area. It enabled me to hear a lot more fresh ideas, to make contact with a lot more people.”

A small number of mental health professionals spoke of being “*knocked back”* from closed meetings or finding out about meetings that they hadn’t been invited to. This sometimes led to disillusionment and lack of engagement.

#### Dynamics of the workshop group

MHPN staff commented that the characteristics of network members had a strong influence on whether networks had formed and continued to meet. Some commented on the level of enthusiasm of members of given initial workshops. As one participant said:

“It can be a bit like speed-dating, and where it works they go ‘yeah, we all want to meet again’ and where it doesn’t work… they are likely to go ‘what are other networks doing?’”

Others noted that having providers who were already networking involved in workshops could act as an enabler by providing networking structures and fostering enthusiasm, but it might have the reverse effect if these providers had pre-existing agendas, factions and/or tensions.

The mix of professionals both at the workshops and in the networks was seen by some MHPN staff and mental health professionals as a key factor that could enable or stifle ongoing networks. On the one hand, many spoke of a desire to have a range of providers involved (particularly GPs), and, on the other hand, a smaller number noted the challenges in dealing with interdisciplinary tensions. Some mental health professionals felt that the workshops were dominated by certain disciplines, and that this led to others feeling under-valued. One social worker commented:

“About five out of ten [GPs] never referred to anyone but psychologists. I actually felt like a non person by the end.”

### Meso-level factors

#### Communication

Mental health professionals and MHPN staff stressed the importance of good communication from MHPN. MHPN staff acknowledged that “*constant communication”* with network members between meetings was crucial. Keeping in contact with members between meetings fosters a sense of belonging to the group and is likely to keep members engaged in the network. Unclear communication was often blamed for groups failing to continue past the first meeting, despite interest from participants in meeting again. This is illustrated by the following comment:

“There just hasn’t been the follow up in my experience … It just starts and doesn’t carry on. I think there could’ve been some improvement there to facilitate [communication] between sessions.”

#### MHPN support

Many staff commented that without the involvement of MHPN the networks would flounder. They saw MHPN’s capacity to provide encouragement and foster a sense of belonging to a larger national enterprise as a motivational force.

On a more practical note, they commented that networks often sought guidance from MHPN regarding network meeting purpose, format and content, and that provision of this guidance was an enabler of network success. As one participant said:

“You are in a really good position to give people examples of what other groups are doing, what’s working.”

Many staff saw MHPN’s role in providing administrative assistance (e.g., communicating with network members, organising meeting venues, managing meeting invitations and acceptances) as key. The following comment typifies participants’ responses:

“Support the networks as much as possible with those small administrative tasks that we can do early on … and be able to hand them over a nice neat package of their network… give them that so then they’ve got time to think about those other more important issues like what they are going to discuss, what are their common purposes …”

However, MHPN staff felt that staffing levels had limited their capacity to provide this kind of support to all groups. As a result, they had to prioritise their work and not all workshop groups and fledgling networks got the same level of support. One staff member noted:

“It’s constant tension between supporting the groups that are rolling … versus engaging with new groups …”

#### Practical resources

Mental health professionals and MHPN staff commented on the practical resources provided by MHPN, particularly MHPN Online. This was seen as important for assisting professionals to become more aware of networks in their area, enabling them to move between groups, and allowing members who might not be able to make it to meetings to participate. As one participant said:

“It has potential to draw on people who at the moment wouldn’t necessarily be engaging with the group, because they wouldn’t necessarily be able to make it face to face.”

#### Macro-level factors

Staff and mental health professionals raised issues regarding the context of the MHPN initiative within Australian primary mental health care funding and culture. Many mental health professionals noted that there was a need for financial support in order for the networks to survive. These mental health professionals argued that financial support was necessary to cover network costs. They also indicated that there was a cost in terms of people’s time, and that reimbursement for this would enhance the likelihood of a network continuing. The importance that these mental health professionals placed on ongoing external funding support is exemplified by the following comment:

“*I’m saying that if you don’t continue to provide a genuine commitment to ongoing support, it won’t be sustainable.”*

Some mental health professionals took this further and considered the context within which primary mental health care services are delivered in Australia. Since the end of 2006, selected services provided by eligible allied health professionals have attracted rebates from Medicare Australia’s universal health insurance system. Certain rules surround the rebates: they are triggered by a standardised referral from a GP, they differ according to who is providing care (e.g., psychologists *versus* social workers) and on the duration of the consultation, and they are only available for certain types of care (e.g., cognitive behavioural therapy). Several mental health professionals felt that this fee-for-service approach was counter to collaboration, making comments like:

“… If we want people to work in a collaborative approach, perhaps the philosophy needs to be underpinned, you know, in some of the rebates.”

Professionals also commented on their reluctance to use their unpaid time to focus on collaborative activities:

“[There is a] gulf between [salaried] public [sector providers] and [fee-for-service] private [sector providers]. The public [providers] have the capacity to sit around and have meaningful conversations and get paid. Private [providers] don’t get paid unless we have patient contact.”

## Discussion

These findings are consistent with previous research that has suggested that collaborative care is made difficult by boundaries between professionals and within health services [4], and that co-ordinated leadership and external support is needed in order to overcome barriers to collaboration [5,6]. While collaborative care can lead to enhanced patient outcomes [1,2], the findings of the MHPN program illustrate that achieving interdisciplinary mental health care is challenging, though perhaps not impossible. The challenges are particularly evident in the primary care context where clinicians most often work in private rooms and are funded purely on a fee-for-service basis. Achievement of interdisciplinary care requires continued and careful attention, and significant effort and change at the level of the individual clinician, within the supporting organization(s) and at also at the wider community and policy level.

The findings from the current evaluation suggest that MHPN’s approach to establishing sustainable interdisciplinary networks for the purpose of promoting collaborative care has been influenced by a number of factors at the micro-, meso- and macro-levels. When reflecting on these different levels we can see the interconnected nature of these key factors in sustainability such as program design and implementation, organizational and community support.

At the micro level, a range of factors had promoted or constrained whether networks had continued to meet. Networks that had persisted in meeting regularly and that were engaging a good mix of professional disciplines tended to be led by well-respected co-ordinators, involve members who are enthusiastic and keen to learn from each other, and have a flexible structure and meet regularly for a well-defined purpose. These ‘champion’ co-ordinators reflect an element of program design and implementation, which the literature suggests is important for program sustainability [14]. At the micro and meso levels, these features are underpinned by good communication between all parties. This has been identified as important in maintaining interdisciplinary collaborations in other contexts [11,19]. Good communication leads to a shared understanding of the ongoing purpose of the network and how collaborative efforts can be fostered, and engenders mutual respect among its membership. Poor communication suggests program design and implementation barriers that prevent networks forming or continuing simply because communication between individuals about future activities has been hampered (micro) or communication with MHPN has been insufficient (meso).

At the meso- and macro-levels, the key issue relates to resourcing. The support and practical resources provided by MHPN have been crucial in guiding successful networks as they form and then continue to meet on a regular basis, with a lack of resources, including MHPN staff levels and online resources, noted as potential barriers. More broadly, at a macro level, however, there appears to be a need for financial support that recompenses mental health professionals for engaging in collaborative activities. Lack of sufficient resources has been observed by others to be a barrier to program sustainability [19], particularly if these resource constraints arise because the intent of the program (in this case, fostering collaboration) does not align well with the broader context within which the program sits (in this case, the fee-for-service Medicare system) [20]. This barrier points to a potential point of tension between individual and organizational goals (sustainable networks) and perhaps the macro- community level context of recognition of activities through financial incentives.

Checkland, Harrison and Marshall (2007) argue that ‘barriers’ reported as preventing change are less important than the context and underlying social relations that have given rise to them, and that consideration of the identity of participants may be key to bringing about change. Their argument is fairly consistent with the findings reported here. Some of the key ‘barriers’ reported by participants related to confusion about how to move forward with interdisciplinary collaboration. These issues could be reframed as difficulties that professionals are experiencing in identifying themselves as interdisciplinary practicing professionals. As mentioned earlier, interdisciplinary care was not previously common in Australia, and is largely not supported by government funding bodies. The change that MPHN is attempting to bring about in the primary care landscape requires a paradigm shift in the way that professionals interact with each other and see themselves. Thus, ultimately the success of MHPN may be less due to its assistance with the logistics of change, and more due to its influence on the beliefs of mental health professionals and the culture supporting interdisciplinary mental health care practice in Australia.

### Strengths and limitations

This study had a number of strengths; most notable is the fact that the evaluation was developed alongside the MHPN project, in collaboration with MHPN. This helped ensure that the findings would be meaningful and relevant to MHPN. The size and scope of the evaluation was also a major strength. Nearly 12,000 professionals took part in MHPN activities over the course of the larger evaluation, which meant that there was a very large pool of potential participants for the focus groups. Participants could be chosen in a selective and stratified manner to ensure representation from all localities and professional groups. However, the sample of participants may also have been a limitation of the study. It is possible that the mental health professionals who took part in MHPN activities were those that were interested and motivated to take part in interdisciplinary collaborative mental health care or those who had strong opinions; this may be even more the case for those professionals who agreed to take part in the focus groups. Thus, the study may not be representative of all mental health professionals. Also, the study of the sustainability of MHPN networks was limited by the timing of the focus groups, which were undertaken during the early stages of network development due to the time constraints of the evaluation.

## Conclusions

This paper provides some insights into the factors that promote and prevent ongoing viability of interdisciplinary networks of mental health professionals and these insights could benefit others trying to achieve similar outcomes within their workplaces, communities, or countries. Further evaluation efforts will be necessary to determine what factors promote or impede sustainability in the long term. These future evaluation activities will need to be quite sophisticated if they are to accurately reflect the achievements of networks.

MHPN has made advances towards the establishment of local networks of mental health professionals, with 81% of initial workshops resulting in networks in less than two years of program establishment. Given that MHPN’s starting point was a situation in which interdisciplinary networking was far from the norm, this is a significant achievement. Several barriers and enablers to the process of creating and sustaining interdisciplinary, collaborative networks have been identified. Continued support for networks is vital, and issues of resourcing and incentives will need to be addressed.

## Competing interest

The evaluation reported in this paper was funded by the Mental Health Professionals Network (MHPN).

## Authors’ contributions

KK, JC, JF & AM were responsible for the day to day management of the evaluation and its reporting. All contributed to the preparation of this manuscript for publication. AN served as evaluation manager during the course of the evaluation and helped to draft the manuscript. JP conceived of the study, and participated in its design and coordination and helped to draft the manuscript. All authors read and approved the final manuscript.
